# The Increased Cost of the Probationer

**Published:** 1920-05-01

**Authors:** 


### THE MATRONS' AND SISTERS' DEPARTMENT.

### THE INCREASED COST OF THE PROBATIONER.

The old contention that hospitals were carried on by means of sweated labour was at no time founded on fact so far as probationers were concerned. Even in the days when probationers paid a premium for admission and went on working for three years before obtaining as much as £20 a year it was easy to demonstrate that the money value of the advantages they received totalled up to a considerable wage. Their real grievance lay, in our judgment, in the conditions of service, which compelled them to wait till the age of twenty-three before commencing their apprenticeship.

Now, however, things have changed to such an extent that the hospitals are paying at a very high rate indeed for totally unskilled apprentices. Their finances are seriously hampered by the enhanced rates of pay, and, what is even more serious, the requirements of the nurse-training schools, outside the question of salaries, become ever more and more costly. The demands of probationers to be treated from the beginning as well-paid workers are steadily killing the goose who lays the golden egg—of free training. Will the hospitals continue to be able to procure money from the public to confer upon women, often indifferently educated, the benefits they are now expected to provide in return for eight hours of unskilled labour a day?

It ought not to be forgotten in estimating the yearly cost to the charity of a probationer that the building and equipment of a modern nurses' home represents a capital charge of some £300 for each nurse, which works out, with repairs and renewals, at some £20 a head for each member of the staff. This is merely the beginning, and is an item that is never reckoned at all in estimates of the nurse's cost. Yet at the present time one hospital after another which is in pressing difficulties has to raise, in addition to the sums required, so to speak, for daily bread, an enormous sum for the enlargement or complete rebuilding of the nurses' home.

The cost of food has gone up quite 50 per cent. in the best-managed institutions, and far beyond this in those which are carelessly conducted. The old estimate of 10s. a week which used to suffice for board must certainly be expanded to 15s., and it may be difficult to prove actual extravagance against housekeepers who exceed this limit. Here, then, is a yearly cost of £39 a head, more or less, and generally more. The domestic expenses, upkeep, crockery, cleaning materials, etc., have, of course, more than doubled in price, and should now reckon at £1 per head. Heating and lighting have increased to an extent which is difficult to estimate, especially as they are to a considerable extent unaffected by fluctuations in numbers. But at present prices a sum of at least £5 per head ought to be reckoned off for fire and light.

The next serious item in the probationer's cost is service. In the nurses' home about one maid is required to each ten nurses, though, as systems vary, this is a rough estimate. Each maid certainly costs not less than £60 a year, so that about £6 a head must be allowed on this score. Laundry has risen appreciably in cost, and now stands for £8, £10, or even £13 per head in the year. A general average of £10 on this score would be fair.

We now come to the expenses of tuition. In former days a probationer scrambled through her course as best she could. She listened to one or two courses of lectures, was talked to about them by one of the sisters, and then went through her examinations, which were not very seriously taken by any one. What is the present cost of running a first-rate school for nurses? Lectures from several medical men, demonstrations and classes to follow these up, outside examiners, class-rooms equipped with expensive models, a good library, cooking lessons, with examination, massage, electricity, perhaps free midwifery training, a hundred precautions in imparting practical instruction in the wards—above all, the exclusive services of a sister-tutor, with possibly an assistant, both receiving good salaries after passing a university course; these are some of the advantages provided for the lucky probationers of to-day. Should we be putting the cost of these privileges enjoyed by the probationer in preparation for her career as a nurse at too high a figure if we estimated it at £15 a year? Only by preparing a number of students could this figure be made to cover expenses.

To recapitulate—the probationer costs as follows: Lodging, £20; board, £39; domestic expenses £1; heat and light, £5; service, £6; laundry, £10; teaching and training, £15. If to this be added the now very usual sum of £20 a year for salary, we reach a total of £116. Some of these items may be reduced by rigid economy. Even so, it would be hard to get below £100 a year.

